# Single-Cell Transcriptomics of Epstein-Barr Virus and Human Herpesvirus 6 Coinfection

**DOI:** 10.1128/mra.00342-23

**Published:** 2023-06-20

**Authors:** JJ L. Miranda

**Affiliations:** a Department of Biology, Barnard College, Columbia University, New York, New York, USA; DOE Joint Genome Institute

## Abstract

Epstein-Barr virus (EBV) and human herpesvirus 6 (HHV-6) infections are widespread in human populations. Here, I describe single-cell RNA sequencing of two lymphoblastoid cell lines harboring both episomal EBV and inherited chromosomally integrated HHV-6. Rare instances of HHV-6 expression appear enriched with EBV reactivation.

## ANNOUNCEMENT

Herpesviruses have ubiquitously spread among humans. Epstein-Barr virus (EBV), a gammaherpesvirus, infects ~90% of the population (1). The circular DNA genome establishes episomal latency (2) to facilitate lifelong persistence. The name “human herpesvirus 6” (HHV-6) collectively refers to two distinct viruses, human betaherpesvirus 6A and human betaherpesvirus 6B (3), which together infect ~70 to 80% of the population (4). HHV-6A and HHV-6B DNA integrates into telomeres (5), and this inherited chromosomally integrated HHV-6 (iciHHV-6) is vertically transmitted to all somatic cells in ~0.1 to 1% of people (6, 7). The widespread prevalence of these viruses predicts frequent coinfections at the cellular level.

Transformation of primary B cells by EBV *in vitro* generates lymphoblastoid cell lines (LCLs) (8) that have been extensively studied as tools for biology research. B cells from some donors contain iciHHV-6 (9). These resulting lines provide a practical opportunity to study the genomics of EBV/HHV6 coinfection.

The EBV-immortalized lines HG00362 and HG01277 (Coriell Institute for Medical Research, Camden, NJ) contain iciHHV-6B and iciHHV-6A, respectively (9). LCLs were cultured under standard conditions (10). Each line was prepared for single-cell RNA sequencing (scRNA-seq) using the Gel bead in EMulsion (GEM) droplet-based system (11) with a Chromium Next GEM Single Cell 3’ GEM, Library & Gel Bead Kit v3.1 (10X Genomics, Pleasanton, CA). Sequencing of libraries from HG00362 and HG01277 cells yielded, respectively, 350 and 300 million paired-end reads of 101 nucleotides.

A pipeline optimized for simultaneously measuring viral and human transcripts (12, 13) was modified for the analysis of EBV and HHV-6. Cell Ranger (10X Genomics) was used to demultiplex raw base call files, trim template switching oligo and poly(A) sequences, align reads, and count unique molecular identifiers (UMIs). Custom references contained the genomes of human GRCh38, EBV B95-8 (GenBank accession number NC_007605.1), and either HHV-6A U1102 (NC_001664.4) or HHV-6B Z29 (NC_000898.1). Viral genomes were appended as extra chromosomes.

**FIG 1:**
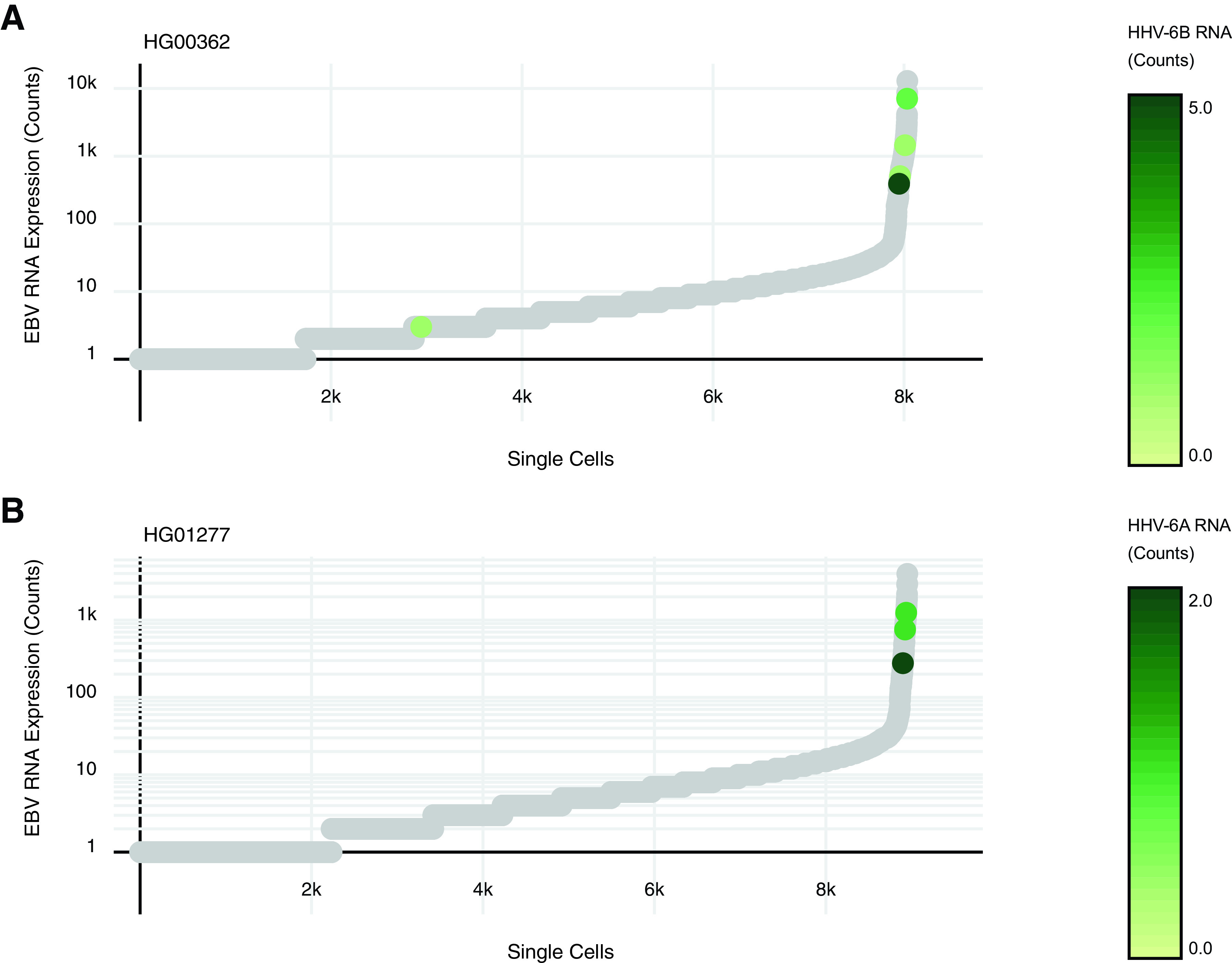
Coexpression of EBV and HHV-6 RNA in single cells. EBV and HHV-6 transcription in the HG00362 (A) and HG01277 (B) LCLs measured by scRNA-seq. Each individual dot on the *x* axis represents a single cell rank-ordered according to EBV RNA levels. Gene expression was measured by the number of UMI counts per cell. EBV transcription is denoted on a log scale on the *y* axis. HHV-6 transcription is depicted by green dots, colored as indicated on the linear-scale heatmap. The gray dots represent cells with no detectable HHV-6 transcription.

HHV-6 RNA is enriched with high levels of EBV gene expression (Fig. 1). The majority of cells in both LCLs display a range of EBV transcription across ~4 orders of magnitude. The upper threshold represents reactivating lytic EBV (14, 15). HHV-6 transcription, on the other hand, is rarely detectable in both LCLs. These scRNA-seq results contrast with those of bulk RNA-seq experiments (16), which could not detect any HHV-6 transcripts in LCLs. The HG00362 data set contains 5 cells with HHV-6B RNA. The HG01277 data set contains 3 cells with HHV-6A RNA. UMI counts fall in the 1 to 5 range, most 1 to 2. With only 1 exception, HHV-6 transcription is found coexpressed with high levels of EBV transcription. This is not related to sequencing depth, as HHV-6 expression is not overrepresented with high levels of human transcription. I call attention to this rare correlation between HHV-6 expression and EBV reactivation while simultaneously encouraging future scRNA-seq studies of EBV/HHV-6 coinfection.

### Data availability.

These data have been deposited in the NCBI Gene Expression Omnibus (17, 18) under accession number GSE230239, which links to NCBI Sequence Read Archive entries SRX20032490 and SRX20032491.
